# Intestinal Transcriptome Analysis Reveals Soy Derivative-Linked Changes in Atlantic Salmon

**DOI:** 10.3389/fimmu.2020.596514

**Published:** 2020-12-11

**Authors:** Viswanath Kiron, Youngjin Park, Prabhugouda Siriyappagouder, Dalia Dahle, Ghana K. Vasanth, Jorge Dias, Jorge M. O. Fernandes, Mette Sørensen, Viviane Verlhac Trichet

**Affiliations:** ^1^ Faculty of Biosciences and Aquaculture, Nord University, Bodø, Norway; ^2^ SPAROS Lda., Olhão, Portugal; ^3^ DSM Nutritional Products, Global Innovation, Kaiseraugst, Switzerland

**Keywords:** aquafeed, *Salmo salar*, soy saponin, intestinal inflammation, flow cytometry

## Abstract

Intestinal inflammation in farmed fish is a non-infectious disease that deserves attention because it is a major issue linked to carnivorous fishes. The current norm is to formulate feeds based on plant-derived substances, and the ingredients that have antinutritional factors are known to cause intestinal inflammation in fishes such as Atlantic salmon. Hence, we studied inflammatory responses in the distal intestine of Atlantic salmon that received a feed rich in soybean derivatives, employing histology, transcriptomic and flow cytometry techniques. The fish fed on soy products had altered intestinal morphology as well as upregulated inflammation-associated genes and aberrated ion transport-linked genes. The enriched pathways for the upregulated genes were among others taurine and hypotaurine metabolism, drug metabolism—cytochrome P450 and steroid biosynthesis. The enriched gene ontology terms belonged to transmembrane transporter- and channel-activities. Furthermore, soybean products altered the immune cell counts; lymphocyte-like cell populations were significantly higher in the whole blood of fish fed soy products than those of control fish. Interestingly, the transcriptome of the head kidney did not reveal any differential gene expression, unlike the observations in the distal intestine. The present study demonstrated that soybean derivatives could evoke marked changes in intestinal transport mechanisms and metabolic pathways, and these responses are likely to have a significant impact on the intestine of Atlantic salmon. Hence, soybean-induced enteritis in Atlantic salmon is an ideal model to investigate the inflammatory responses at the cellular and molecular levels.

## Introduction

The quality of high-value farmed fishes such as Atlantic salmon (*Salmo salar*) depends to a great extent on the ingredients in their feeds. Furthermore, the increase in the demand for farmed salmon necessitates intensive farming and the adoption of sustainable feed ingredients. Awareness about sustainability and limited availability of finite marine sources has led to the replacement of fishmeal and fish oil in aquafeeds with plant derivatives. The current norm is to incorporate considerable amounts of plant-derived components—proteins from pea, soybean, horse beans, oil from rapeseed, starch and gluten from wheat and maize gluten—in aquafeeds (1). However, carnivorous fishes are known to develop intestinal inflammation, *e.g.* when they consume high levels of terrestrial plant components such as the products from soybean. This undesirable health condition is mainly caused by antinutritional factors in soybean such as soyasaponin, *β*-conglycinin and glycinin (2–4).

Feeding fish with high levels of full-fat soybean products or low levels of the soybean meal with other legumes is known to not only affect their growth and nutrient utilization but also can disturb the integrity of the distal intestine. Plant-derived ingredients may shift the intestinal microbial community composition (5, 6), which in turn can affect the overall intestinal health, including immunity (7). Inflammation is the first sign of intolerance to dietary components, and fishes that develop the non-infectious disease will have widened lamina propria with many inflammatory cells, less absorptive vacuoles, and shortened brush border microvilli in their distal intestine (4). Fish with inflamed distal intestine will be characterized by poor nutrient digestibility and disturbances in transcellular water transport, especially when their feeds contain >30% soybean meal (8–10). Furthermore, the combination of soybean (even defatted meal) and other legumes can affect the metabolism and gut functions in Atlantic salmon; aberrations in epithelial barrier and major transcriptome changes are already reported (11–13). Studies that employed other fish species have also indicated the adverse effects of soybean meal or soy saponins on growth, nutrient utilization, antioxidant status, and intestinal morphology (14, 15). Thus, it is evident that plant-derived ingredients with antinutritional factors can affect the health of farmed fish, leading to undesirable fish welfare issues. Although the aforementioned studies have described soy-related inflammation in Atlantic salmon, it is imperative to further understand the molecular changes in the intestine of fish that develop intestinal inflammation. Identification of appropriate markers of inflammation would not only enable effective screening of new feed ingredients, but also enhance our understanding of the processes that are affected during inflammation in a lower vertebrate.

We employed a well-studied fish model, Atlantic salmon, to examine the dietary soy products-linked disturbances in the intestine. This study describes the changes in micromorphology, transcriptome of the distal intestine, and whole blood cells in the inflammation (SO) group compared to the control (CO) group. The novelty of the current study lies in the adoption of RNA-Seq to clearly delineate the molecular changes evoked during inflammation.

## Materials and Methods

### Experimental Design

In this study, we examined the intestinal transcriptome of a carnivorous fish that developed inflammation. For this, Atlantic salmon post-smolts procured from a local commercial producer (Sundsfjord Smolt, Nygårdsjøen, Norway) were maintained at the Research Station of Nord University, Bodø, Norway, for 4 months. For the experiment, 120 fish (128.66 ± 13.29 g; mean ± SD) were randomly distributed to replicate tanks of the two study groups, *i.e.* CO and SO groups. During a 2-week acclimation period, all the fish were fed a commercial feed (Ewos AS, Bergen, Norway). The 800 L tanks were part of a flow-through seawater system at the Research Station. Water from a depth of 250 m in Saltenfjorden was pumped, filtered, and aerated, and then used for rearing the fish. The water flow rate was maintained at 1,000 L per h, and the average temperature and salinity of the rearing water were 7.6°C and 34 g L^−1^, respectively. The dissolved oxygen saturation values, measured at the water outlet of the tanks was in the range 87–92%, and throughout the experimental period, we employed a 24-h light photoperiod.

The two feeds for the study were prepared as 3 mm extruded pellets by SPAROS Lda (Olhão, Portugal); a control feed for the CO group, and a soybean and soy saponin-containing feed for the SO group (**Supplementary Table 1**). The feeds contained only around 25% ingredients that were of marine origin; 15% fishmeal and 9.4–9.6% sardine oil. All other ingredients were from different plants: soybean, wheat, corn, and rapeseed. Although both the feeds contained soy protein concentrate and soy lecithin powder, only the SO feed contained soybean meal, soybean meal full fat and soy saponins (40% purity). The latter two ingredients were included in the SO feed to induce distal intestinal inflammation in the fish. These feeds were fed *ad libitum* to the respective fish groups, using automatic feeders (Arvo Tech, Huutokoshi, Finland). The feeders delivered the feeds twice a day, 08:00–09:00 and 14:00–15:00, and the daily feeding rate was 1.2% of the fish weight.

### Sampling

After 36 days of feeding, the fish were euthanized with an overdose (160 mg L^−1^) of MS222 tricaine methanesulfonate (Argent Chemical Laboratories, Redmond, WA, USA) to collect the blood, distal intestine, and head kidney. The distal segment (DI) of the intestine was chosen as it is affected by dietary soy products. On the other hand, the head kidney (HK) was selected because it is a key organ involved in the systemic immune responses. First, 2 ml of whole blood (WB) was drawn from *vena caudalis* of fish (n = 10) using heparinized syringe and transferred to 15 ml centrifuge tubes containing 4 ml of culture medium (described later). Thereafter, the fish were dissected under aseptic conditions to collect the DI (after removing the contents) and HK samples (n = 6) which were then transferred to cryotubes, snap-frozen in liquid nitrogen and stored at −80°C. In addition, we had collected the DI samples on day 4 (n = 6); this sample was employed only to understand the histological changes at an early time point. The anterior most portion of the DI segment collected on day 36 was also used for the histology study. DI obtained on day 36 was used for the RNA-Seq and qPCR studies, and HK was employed for the RNA-Seq.

### Intestine Tissue Histomorphology

To understand the morphological changes in the distal intestine, samples of the segment were processed, and 5 μm sections of the tissues from the CO and SO groups were prepared as reported in Vasanth et al. (16). Alcian Blue-Periodic Acid Schiff’s reagent (AB-PAS, pH 1.0) (17) was used to stain the sections for mucins. Thereafter, the sections were viewed using a microscope (Olympus BX51, Olympus Europa GmbH, Hamburg, Germany) with a maximum magnification of 200×. The photomicrographs were captured employing a Camera (SC180, Olympus) and processed with the imaging software CellEntry (Soft Imaging System GmbH, Munster, Germany).

### Intestine/Head Kidney Transcriptome—RNA Isolation, Library Preparation, and Sequencing

To delineate the changes of the respective transcriptomes, total RNA was extracted from DI and HK samples following the QIAzol protocol (Qiagen, Hilden, Germany). RNA purity and quantity were determined using the NanoDrop 1000 (Thermo Fisher Scientific, Waltham, MA, USA). Furthermore, the integrity of the RNA isolated from the two organs was assessed using Agilent RNA screen tapes, following manufacturer’s protocol, on the 2200 TapeStation system (Agilent Technologies, Santa Clara, CA, USA). Only samples with RIN > 7.5 were used for library preparation.

RNA sequencing libraries were prepared according to the protocols of Siriyappagouder et al. (18) by using NEBNext ultra II directional RNA library preparation kit with poly (A) mRNA magnetic isolation module (NEB #E7490; New England BioLabs^®^, Herts, UK). Briefly, 1 µg of total RNA was used as the starting material for the library preparation. The mRNA was enriched using oligo-dT magnetic beads and fragmented to ∼100–200 nt, prior to synthesis of the first and second cDNA strands. The resulting cDNA was purified and 3′ end repaired for adapter ligation. Further PCR enrichment (8 cycles) was performed, and PCR products were cleaned with AMPure XP beads (Beckman Coulter Inc., Brea, CA, USA) to ensure that the libraries were free from residual adapter dimers and unwanted (smaller) fragments. In total, 24 libraries were prepared (12 for DI and 12 for HK); there were six replicates per treatment group. Individual libraries were quantified, normalized and pooled at equimolar ratio and sequenced as single-end reads (75 bp) on an illumina NextSeq 500 sequencer (illumina, San Diego, CA, USA) with NextSeq 500/550 high output v2 reagents kit (illumina). Libraries from the DI and HK samples were sequenced separately by using two flow cells. The obtained raw sequencing data was deposited in the Sequence Read Archive, National Center for Biotechnology Information (NCBI) database under the accession number PRJNA640734.

### Intestine/Head Kidney Transcriptome—Data Processing and Statistical Analyses

Adapter sequences from the raw reads were removed using cutadapt (version 1.12) (19), employing the following parameters: -q 25, 20 –quality-base = 33 –trim-n -m 20. The quality of the clean reads was further assessed using FastQC (Andrews, 2010) and reads with quality <30 were removed. Reference genome of Atlantic salmon (assembly ICSASG_v2) and gene model annotation files were downloaded from NCBI to annotate the sequences. The software STAR (version 2.5.3a) was used to build the index, and cleaned reads were mapped to reference genome with default parameters.

We employed DESeq2 version 1.22.2, which uses shrinkage estimates for both dispersion and fold change to identify the differentially expressed genes (DEGs) (20). An organism database with Entrez geneID was prepared using AnnotationHub version 2.14.5 (21). Pathway enrichment and gene ontology (GO) over-representation of DEGs were assessed with clusterProfiler version 3.10.1 (22). Furthermore, the association of the enriched objects was delineated using the same package. Based on the report by Hong et al. (23), we performed separate enrichment analyses for up- and downregulated genes. The functions of the packages ggplot2 version 3.1.1 (24) and ggraph (25) were used to format the graphs.

### Intestine Transcriptome—Verifying the Expression of DEGs by Real-Time PCR

qPCR was performed to verify the mRNA levels of selected DEGs identified from the RNA-Seq study; here we employed the same samples that were used for the RNA-Seq study. Briefly, 1 µg of total RNA from each sample was reverse transcribed using the QuantiTect reverse transcription kit (Qiagen), according to the manufacturer’s instructions. The obtained cDNA was further diluted 10 times with nuclease free water and used as PCR template. The PCR reactions were conducted using the SYBR green in LightCycler^®^ 96 Real-Time PCR System (Roche Holding AG, Basel, Switzerland), following the method previously described by Vasanth et al. (16). The reactions were performed in duplicate on samples from six fish per group.

Primers for the selected genes were designed using the Primer-BLAST tool in NCBI. The primer secondary structures such as hairpin, repeats, self and cross dimer were accessed with NetPrimer (Premier Biosoft, Palo Alto, USA). The primers for the reference and target genes are given in **Table 1**. Using geNorm (26), a geometric normalization factor was computed for each of the samples based on the relative quantities of the two most stable genes (*rps29* and *ubi*) from among the set of four reference genes—elongation factor 1AB (*ef1ab*), ribosomal protein L13 (*rpl13*), ribosomal protein S29 (*rps29*), and ubiquitin (*ubi*). The expression levels of all the target genes were then calculated relative to the normalization factor.

**Table 1:** Details of primers used for the qPCR verification study.

Gene	Primer sequence	PCR efficiency (%)	Amplicon size (bp)	GenBank accession numbers
*anxa2*	CATTGCAGAAAGAATACAAAGGGG-**F**	92.1	96	XM_014161401.1
	CCAGCGTGACAATACTGTG-**R**			
*cath1b*	GTCCTCTGAAGAAAAATGGGAAAC-**F**	88.5	135	NM_001123586.1
	GCATAGCATCTTCTGCCTC-**R**			
*cath2*	CCGATTCTGGAGACTGGCAA-**F**	96.5	111	NM_001123573.1
	TGTCCGAATCTTCTGAGTGC-**R**			
*clcn1*	TCAGCAACAACAGTCTCT-**F**	93.0	82	XM_014152555.1
	GCTGTGGATGGTGCTGTT-**R**			
*clcn2*	CTCGGACACATCAGTAAG-**F**	90.8	123	XM_014184291.1
	TGAGGGAGGTGGAGTCTAGC-**R**			
*csad*	CGGTCTGGCTGACATAAT-**F**	88.2	127	NC_027317.1
	AGTTGACTCGTCCACCCTGA-**R**			
*gal3*	CGGAGCTACTAACAGATA-**F**	90.1	127	NM_001140833.1
	GTTGGCTGGTTGGGTTGC-**R**			
*gsto1*	GCTTCATGCCAAGGGGAT-**F**	89.9	107	NM_001141472.1
	TCTCCAATGTCGGAACCAGG-**R**			
*lysc2*	ATGAGAGCTGTTGTTGTTC-**F**	97.0	144	XM_014145497.1
	AGACAGGCACACCCAGTT-**R**			
*mta*	TGAATGCTCCAAAACTGG-**F**	88.3	130	NM_001123677.1
	CCTGAGGCACACTTGCTG-**R**			
*rbp2*	GACCTGCTACACCTGGACATC-**F**	104.0	147	NM_001146482.1
	TCTCAACTGGCCTACCTG-**R**			
*slc26a6*	TGGGCATGGAACACCTGA-**F**	91.1	124	NC_027312.1
	CACCAACTGTTAAACTCG-**R**			
*slc6a19*	ATGGAGGAGGAGCGTTTA-**F**	100.5	158	NM_001141815.1
	CGATGCCAACACCTGTCAGA-**R**			
*slc6a6*	GGTGTAATTCATTTCCGATGC-**F**	95.8	109	XM_014134772.1
	CTCTTTCTGTGCCATGCTGC-**R**			
*tnfrsf1b*	TCGGAGGTGTTATCGGAG-**F**	91.7	80	XM_014133111.1
	CCTGGACCCTGTGAAGACTTT-**R**			
Reference genes				
*ef1ab*	TGCCCCTCCAGGATGTCTAC-**F**	94.5	59	BG933853
	CACGGCCCACAGGTACTG-**R**			
*rpl13*	CGCTCCAAGCTCATCCTCTTCCC-**F**	95.5	79	BT048949.1
	CCATCTTGAGTTCCTCCTCAGTGC- **R**			
*rps29*	GGGTCATCAGCAGCTCTATTGG-**F**	93.3	167	BT043522.1
	AGTCCAGCTTAACAAAGCCGATG-**R**			
*ubi*	AGCTGGCCCAGAAGTACAACTGTG-**F**	94.9	162	AB036060.1
	CCACAAAAAGCACCAAGCCAAC-**R**			

Annexin 2-like (anxa2), cathelicidin 1-B (cath1b), cathelicidin 2 (cath2), chloride channel protein 1-like (clcn1), chloride channel protein 2-like (clcn2), cysteine sulfinic acid decarboxylase-like (csad), galectin-3 (gal3), glutathione S-transferase omega-1(gsto1), lysozyme C II (lysc2), metallothionein A (mta), retinol binding protein II (rbp2), solute carrier family 26 member 6-like (slc26a6), solute carrier family 6 member 19 (slc6a19), solute carrier family 6 (neurotransmitter transporter, taurine) member 6 (slc6a6), and tumor necrosis factor receptor superfamily member 1B-like (tnfrsf1b).

### Cytological Studies—Cell Isolation and Culture

To further understand the feed-induced inflammatory responses, we examined the immune cell population in WB. The cell isolation and culture procedures for the WB samples have been described in Park et al. (27). Briefly, the collected WB was kept in 4 ml of ice-cold L-15 medium. To isolate the WB leucocytes (WBLs), we employed Percoll (Sigma-Aldrich, Oslo, Norway) 40%/60%. After centrifugation (500 × *g*, 30 min, 4°C), the cells that were separated at the interface of the Percoll gradients were carefully collected and washed twice with 4 ml of ice-cold L-15 by centrifugation (500 × *g*, 5 min, 4°C).

### Cytological Studies—Flow Cytometric Assay

ImageStream^®X^ Mk II Imaging Flow Cytometer (Luminex Corporation, Austin, TX, USA) equipped with two argon-ion lasers (488 and 642 nm) and a side scatter laser (785 nm) was used for the flow cytometric assays. The acquired cell data was analyzed using IDEAS 6.1.822.0 software (Luminex Corporation). The protocols for the flow cytometric assays were previously described in Park et al. (27).

### WB Lymphocyte-Like Cell Population

To compare the percentage of lymphocyte-like cells of fish from the CO and SO groups, aliquots containing 1 × 10^6^ cells of WBL in 50 µl PBS were prepared in 1.5 ml microcentrifuge tubes. Before every sample was run through the flow cytometer, 1 µl of propidium iodide (PI; 1 mg/ml, Sigma-Aldrich) was added to stain dead cells. Next, 1 mW 488 nm argon-ion laser and 0.47 mW 785 nm side scatter laser in the imaging flow cytometer were set to detect the dead cells (702/86 nm bandpass; Channel 5) and cell complexity (772/55 nm bandpass; Channel 6), respectively. Cell analyses were performed on 10,000 cells acquired at low speed (300 objects/s) and at a magnification of 40×. Dead cells were estimated as the percent of cells that were positive for PI (red fluorescent cells). After excluding the dead cells, viable cells were analyzed by generating brightfield (BF) area (size) *vs*. side scatter (SSC) intensity (complexity) dot plots. The settings of the cytometer were kept identical during the analysis of all the samples. We adopted a gating strategy based on our IFC protocols (27); using HK IgM^+^ cells isolated by magnetic-activated cell sorting, lymphocyte localization (low BF area and low SSC intensity) was determined employing a BF area *vs*. SSC intensity plot. In the present study, the percentages of cells in the lymphocyte localization gates were compared to determine the differences in the CO and SO groups.

### Data Handling and Statistical Analyses—Flow Cytometry and qPCR Studies

The data analyses were performed in RStudio. Normality of the qPCR and flow cytometry data was tested by Shapiro–Wilk Test, and the assumption of equal variance was checked by the Bartlett’s Test. Unpaired Student’s t-test was employed to compare the statistically significant difference between the two groups. Mann–Whitney U test was used for non-parametric data. The differences were considered significant at p < 0.05.

## Results

### Soybean Products Induced Inflammation in the Distal Intestine of Atlantic Salmon

**Figure 1:**
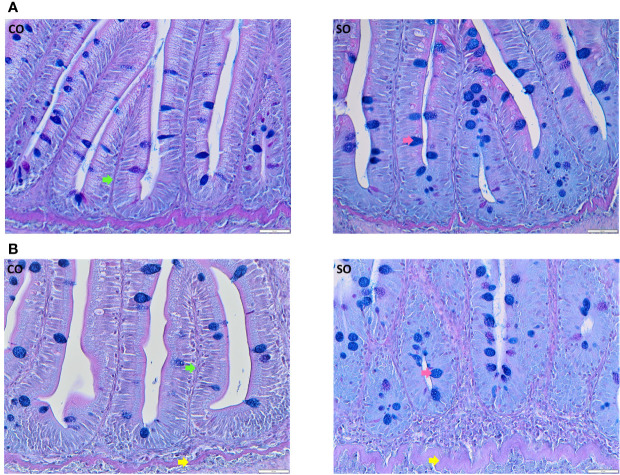
Photomicrographs of the distal intestine of Atlantic salmon from the CO and SO groups. **(A)** Inflammatory features are not visible in the SO group on day 4 after the start of the feeding trial. **(B)** Saponin-induced inflammatory characteristics are evident at day 36 of the trial. Control group—CO, and soy-derivatives fed group—SO. Pink arrow—goblet cells, green arrow: lamina propria, yellow arrow—stratum compactum. Scale bar: 50 µm.

On the 4^th^ day after the start of the trial, the DI of the SO group did not exhibit any characteristic morphological changes associated with inflammation (**Figure 1A**, **Supplementary Figure 1A**). However, at this time point, we could observe a reduction in absorptive vacuoles and more intraepithelial lymphocytes compared to those in the CO group. The morphological differences in the SO group were clearly discernible on the 36^th^ day (**Figure 1B**, **Supplementary Figure 1B**); the SO group had fused villi, fewer supranuclear vacuoles, widened lamina propria, infiltration of inflammatory cells, and enlarged stratum compactum.

### Transcriptome of the Distal Intestine Revealed DEGs Induced by the Soybean Products

The transcriptomes of DI and HK were collected on day 36 when the fish intestine exhibited distinct features of inflammation. A total of over 563 million cleaned reads from 24 (12 from distal intestine and 12 from head kidney) samples were obtained after adapter trimming and quality filtering; of these over 435 million reads were mapped to Atlantic salmon transcriptome and genome. However, one biological replicate of the DI from the CO group was removed from the downstream analysis due to lower (22.83%) mapping percentage (**Supplementary Table 2**). An illustration of the bioinformatics pipeline is provided in **Supplementary Figure 2**.

**Figure 2:**
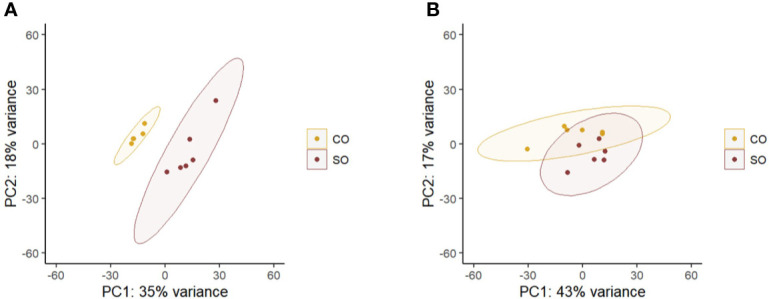
Principal component analysis plot shows the clustering of the CO and SO fish groups. **(A)** The distal intestine and **(B)** head kidney. Control group—CO, and soy-derivatives fed group—SO. Note that from the intestine data, one replicate was removed from the CO group because the reads did not yield a good mapping result.

**Figure 3:**
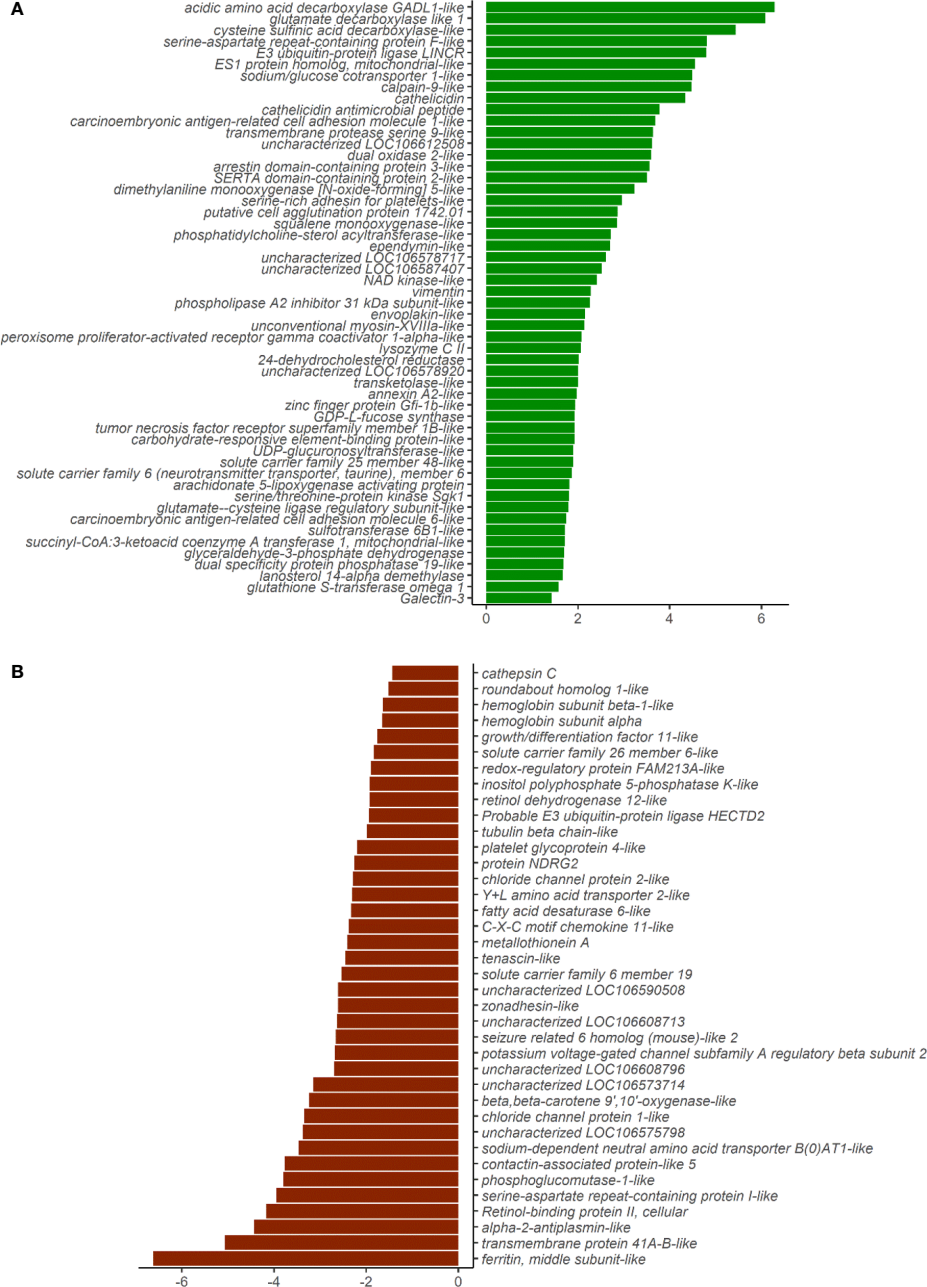
Plot showing the differentially expressed genes in the distal intestine of Atlantic salmon. **(A)** Fifty-three genes were upregulated in the SO group (green bars). **(B)** Thirty-eight genes were downregulated in the SO group (red bars). X-axis labels show the log2foldchange, and the y-axis shows the differentially expressed genes.

The principal component analyses of the normalized counts pointed to the differential clustering of the CO and SO groups in the DI (**Figure 2A**), but not in the HK (**Figure 2B**). The dispersions of the gene expression data, as expected, decreased with increasing mean, and the MA (minus over average) plot revealed the differentially expressed genes after shrinking the dispersions and logarithmic fold changes (DI: **Supplementary Figures 3A, B**; HK: **Supplementary Figures 4A, B**). We identified 53 upregulated and 38 downregulated genes in the DI at a logarithmic fold change threshold of 0.75 and an adjusted p-value of 0.05 (**Figures 3A, B**; **Supplementary Figure 5**). However, DEGs were not detected in the case of HK (p > 0.05).

The upregulated genes in the DI included inflammation-associated genes such as *cathelicidins*, *galectin*, *tumor necrosis factor receptor superfamily member 1B-like*, *lysozyme CII*, *annexin A2*-*like* (**Supplementary Tables 3**, **5**). Some solute carrier families were upregulated while others were downregulated in the SO group (**Supplementary Tables 4**, **5**). Genes connected to chloride channel proteins (*clcn1*, *clcn2*) were downregulated in the SO group. In addition, sodium-associated transporters were altered in the same group (*sodium/glucose co-transporter 1-like, slc5a1*, and *sodium- and chloride-dependent taurine transporter*, *slc6a6* were upregulated and *sodium-dependent neutral amino acid transporter B(0)AT1-like, slc6a19* and *potassium voltage-gated channel subfamily A regulatory beta subunit 2*, *kcnab2* were downregulated). Another anion exchanger, *solute carrier family 26 member 6*, *slc26a6* and the mitochondrial amino acid transporter, *slc25a48* were upregulated in the SO group.

The expression of 15 DEGs was verified by qPCR. The bar plots in **Supplementary Figure 6** show the mRNA levels of the selected genes. The mRNA levels correlated positively with the read counts from the RNA-Seq study (**Supplementary Figure 7**).

### Soybean Products Affected the Biological Pathways

**Figure 4:**
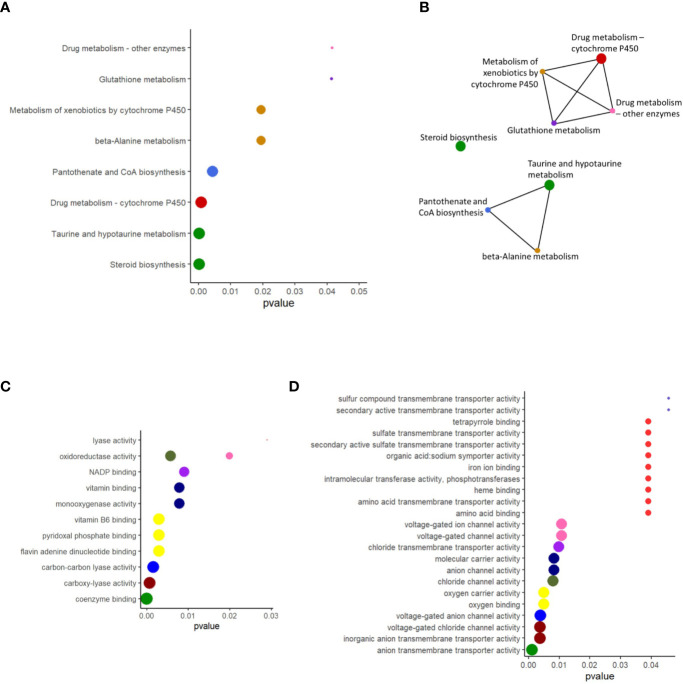
Enriched KEGG pathways and overrepresented GO terms for the upregulated and downregulated genes in the distal intestine of Atlantic salmon. **(A)** The biologically relevant pathways for the upregulated genes were extracted at a p value cutoff of 0.05 for the enrichment tests. **(B)** Network showing the connections between enriched pathways that have overlapping gene sets. **(C)** The biologically relevant GO terms for the upregulated genes were extracted at a p value cutoff of 0.05 for the enrichment tests. **(D)** The biologically relevant GO terms for the downregulated genes were extracted at a p value cutoff of 0.05 for the enrichment tests. X-axis shows the adjusted p values associated with the pathways/GO terms. The pathways shown in panels A and B are color coded similarly. In each figure, the sizes of the dots decrease with the p value, and the colors for dots with same p value are kept the same.

The enriched pathways for the upregulated genes were taurine and hypotaurine metabolism, beta-alanine metabolism, pantothenate and coenzyme (CoA) biosynthesis, drug metabolism–cytochrome P450, drug metabolism–other enzymes, metabolism of xenobiotics by cytochrome P450, steroid biosynthesis, and glutathione metabolism (**Figure 4A**). All these enriched pathways had common genes; the exception was steroid biosynthesis (**Figure 4B**). The package clusterProfiler did not detect any enriched pathways for the downregulated genes. The enriched GO terms based on the upregulated genes were among others, oxidoreductase activity, some binding and lyase activities (**Figure 4C**). More GO terms based on the downregulated genes were enriched; many belonged to transmembrane transporter activity, channel activity and some binding activity (**Figure 4D**). For the upregulated genes, NADP, vitamins, pyridoxal phosphate, flavin adenine dinucleotide, and coenzyme binding were enriched (**Figure 4C**). On the other hand, for the downregulated genes, tetrapyrrole, iron ion, oxygen, heme, and amino acid binding were the enriched GO terms (**Figure 4D**).

### Soybean Products Induced Inflammation Was Marked by an Increase in WB Lymphocyte-Like Cells

**Figure 5:**
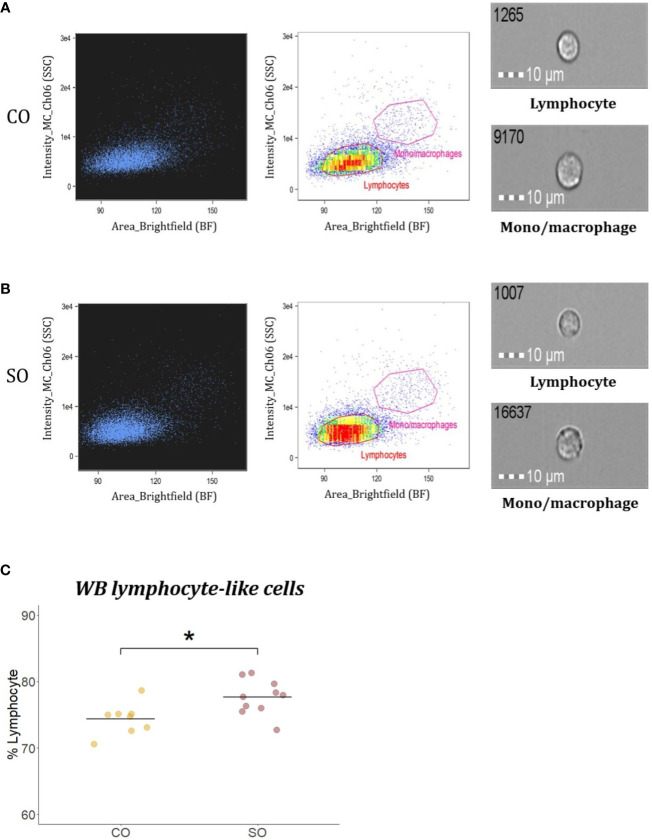
Flow cytometric analysis of WB lymphocyte-like cells from Atlantic salmon. Brightfield (BF) area (x axis; cell size) *vs* side scatter (SSC) intensity (y axis; cell complexity) plot shows WB leukocyte population from fish (n = 10) fed **(A)** CO or **(B)** SO feeds. Representative images of cells captured with 40× objective from each gate are shown. Scale bar = 10 µm. **(C)** Plot showing the percentage of WB lymphocyte-like cells from fish (n = 10) from the two groups. The 10 dots are values from the 10 fish. The differences were considered significant at p < 0.05, indicated with an asterisk.

To compare the percentage of WB lymphocyte-like cells from the CO and SO groups, WBL population was presented on a brightfield area (cell size) *vs* side scatter intensity (cell complexity) dot plot (**Figures 5A, B**). The gate for lymphocyte-like cells was determined based on salmon IgM^+^ cell area, as previously described by Park et al. (27). From **Figure 5C**, it is evident that the SO group (77.64%) had higher percentage of WBL-like cells compared to the CO group (74.35%; p < 0.05).

## Discussion

Diet-induced intestinal inflammation is a common clinical issue that has to be tackled by understanding the associated mechanisms because the disease has pervaded all classes of the global population. In humans, Crohn’s disease and ulcerative colitis are inflammatory bowel diseases. These non-communicable diseases are assumed to be early adulthood diseases caused by genetic and environmental factors which disturb immunity and weaken epithelial barrier (28). Like humans, carnivorous farmed fish are prone to diet-linked allergies that can affect their growth (4, 8–10). Here we describe the soybean/soy saponin-induced inflammation primarily based on the changes in the transcriptome of the DI of Atlantic salmon. The results indicate the alteration of around 90 genes and disturbance of associated pathways and GO terms in the DI of fish. Furthermore, the differential counts of the immune cells also point to possible changes in the immune status of this fish group.

### High Fat Soybean Products and Soy Saponin Induced Inflammation in the Distal Intestine of Atlantic Salmon

Certain soybean products are known to cause inflammation in the intestine of carnivorous fishes (2, 4, 9, 12, 14). High levels of soybean meal in diets or the presence of antinutritional factors is reported to be the main reasons for the inflammatory condition (2, 10). Feeding even 10% of solvent extracted soybean meal or 25% defatted soybean meal can give rise to pathophysiological changes in Atlantic salmon (12, 29). Furthermore, the severity of inflammation in the DI of this fish is dependent on the content of the antinutritional factor (30). A study points out that the impact of soy saponin concentrate (of 69% purity) feeding for 53 days is relatively benign in terms of inflammatory signs (12). In the present study, 36 days of feeding soy products (meal, full fat, and soy saponin) led to the development of inflammation in the DI of salmon. The inflammation characteristics were similar to that described previously by others (4, 12, 30) but not as intense as reported earlier in a chemically-induced inflammation model that employed a direct application method (16).

### Distinct Effects of Soy Products in the Intestine Transcriptome

In this study, in addition to local responses to soybean products in the DI, we anticipated that changes may occur in the HK, a key immune organ. Most transcriptome studies focusing on HK have registered responses to bacterial/viral pathogens or parasites, while some studies have shown that functional feeds could alter expression of genes in the organ, as described in a review article (31). In the present study, we did not detect any DEGs in the head kidney similar to the transcriptome investigation by Tacchi et al. (32) that compared marine protein- and plant protein-based diets; no common genes were expressed in the intestine, muscle, and liver. Diet-induced inflammation may affect systemic response as reported for salmon fed soybean based on 44 common genes expressed in the intestine and liver (33). Furthermore, diets did not alter the HK transcripts in fish, but the responses became evident 24 h after injection with the viral mimic polyriboinosinic polyribocytidylic acid (34). In the present study, we did not find a significant impact of the soy products on the HK transcriptome. The lack of response in HK seems to indicate that it is not the optimal organ to check the diet-induced changes in transcriptome. However, future studies should consider the changes in the liver transcriptome, a metabolically active organ.

### Soy Products Evoked Inflammation-Associated Signals in the Intestine

Bacterial entry through a breach in the epithelial barrier due to defective functions and aberrant innate immune responses are accountable for evoking intestinal inflammation in humans (35). Such an inflammation triggers the transcriptional activation of selected immune-related genes (36).

Cathelicidin genes, *cathl1* and *cathl2*, are known to be expressed in the DI of salmon and were stimulated by pathogenic bacteria; in our study they were upregulated in the DI of fish from the SO group (**Supplementary Figure 6**). Human cathelicidin, LL-37 mRNA, was increased significantly in the inflamed mucosa of ulcerative colitis and Crohn’s disease (37). In addition, it is interesting to note that both *lysozyme CII* and *tumor necrosis factor receptor superfamily member 1B-like* were upregulated. Based on mammalian studies, it is known that pathogen associated molecular patterns are recognized by toll-like receptors that activate the MyD88-independent pathway including the TNF (Tumor necrosis factor) receptor associated factor 6 (TRAF6), which is associated with TNF receptor superfamily (38). Tafalla and Granja (39) have reviewed the involvement of mammalian TNF receptor superfamily 1B (TNFRSF1B) in human T cell activation, and they have suggested the same function in fishes. Another pro-inflammatory mediator, Galectin 3 (40), was altered in inflamed tissues of humans (41), as reported here. A study on striped murrel *Channa striatus* revealed the ability of a galectin, CsGal-1, to agglutinate Gram-negative bacteria (42). Galectin-3 in mammals is known to recognize ‘self’ glycans generally but can interact with the lipopolysaccharide (LPS) of bacteria (43). Galectin-3 is secreted by inflammatory macrophages, and its induced secretion is perceived as a danger signal by the innate immune system (44, 45). The intestinal epithelial breach in the SO group, probably resulting from the unwanted reaction, may have possibly facilitated the entry of bacteria through the mucosal barriers and altered the expression of *gal3.*

Metallothionein is an anti-inflammatory mediator. After LPS treatment, metallothionein knock-out mice showed inflammatory responses in the lung epithelial cells suggesting that metallothionein is involved in cytoprotection during LPS-related inflammation (46). In the present study, metallothionein A was downregulated in the SO group, indicating a possible connection between metallothionein A and intestinal inflammation.

Retinol-binding protein transports vitamin A from the liver to other tissues like the intestine (47). It has been reported that tissue injury during inflammation causes a reduction in the synthesis of retinol-binding protein (48). In our study, the mRNA level of retinol binding protein II in the SO group was significantly downregulated. Notably, a study on humans reported that its concentration in serum decreases during the acute-phase stage of inflammation (49).

As observed in the salmon intestine, the mRNA levels of Annexin A2 were higher in the inflamed mucosa of patients with Crohn’s disease (50). Intestinal epithelial cell turnover prevents pathogen colonization (51), and the actin-binding protein, Annexin A2, is necessary for the movement of intestinal epithelial cells during wound resealing (52). A link between *anxa1* and gastroprotection was suggested in an earlier study on Atlantic salmon (16).

### Soy Products Also Impaired the Transport Mechanisms and Metabolism in the Intestine

Intestinal junctional molecules play key roles in sustaining epithelial integrity (35). Dysregulation of epithelial transport or entry of opportunistic microbes through the breaches in epithelial barrier can cause intestinal inflammation (51). The characteristic selective permeability of the epithelial barrier to ions, through gating of ion channels, enables directional transport of ions (53). Chloride channel proteins form gated pores for the passage of anions, and this process is dependent on transmembrane voltage, pH, cell-swelling and phosphorylation (54). The Cl^−^ exits basolaterally, among other means, *via* K^+^/Cl^−^ cotransport, Cl^−^ channel (55), and genes associated with chloride channel proteins were downregulated in the fish fed the soy products. In addition, solute carrier families are involved in ion transport across the apical membrane of fish intestine (55). *Slc6a6* is a sodium and chloride-ion dependent transporter that transports taurine and beta-alanine *via* intestinal brush border (56), and this gene was upregulated in the SO group, and the associated pathways that were enriched were taurine and beta-alanine pathways. Furthermore, Cl^−^/HCO3^−^ exchange and active transport of most neutral amino acids at the apical membrane of the fish intestine are performed by *slc26a6* and *slc6a19*, respectively (55). These solute carrier families were downregulated in salmon that were prone to the unwanted reaction. Furthermore, the enriched GO terms included chloride channel activity, voltage-gated channel activity, voltage-gated anion activity, anion channel activity *etc*. We also observed a dysregulation in other activities, *viz*. transmembrane transporter activity and symporter activity in the SO group. In addition, *slc5a1*, the key mediator of glucose and galactose uptake from the lumen, and *slc25a48*, mitochondrial amino acid transporter were upregulated, and *kcnab2* (*Potassium voltage-gated channel subfamily A regulatory beta subunit 2*), which has a role in electrolyte transport, was downregulated in the SO group. Such aberrations in humans can lead to irregular nutrient absorption (57) and disturbances in the luminal fluid microenvironment (53). The alterations in iron ion, oxygen, heme, and amino acid binding GO terms, based on the downregulated genes, and NADP, vitamins, and coenzyme binding, based on upregulated genes, also point to the impaired transport mechanisms across the epithelial layer in the SO group. Other studies have also reported the effect of soybean feeding on transporter proteins, which eventually may have a bearing on the transport of nutrients *via* epithelia. In one of them, the soybean meal–wheat gluten combination affected the expression of aquaporins, ion transporters, tight junction, and adherens junction proteins in the DI of Atlantic salmon, although the genes associated with chloride channels remained unchanged (10). Furthermore, Atlantic salmon fed soy saponins alone and soy saponin in combination with soybean meal had aberrations in intestinal permeability (12). The transcriptional changes of the genes linked to the transporter proteins may be indicating disturbances in the transport of essential nutrients and the associated pathways.

Although we identified some enriched pathways linked to the upregulated genes, there was not much overlap in the pathways we obtained and those reported previously. Based on current as well as earlier studies, steroid biosynthesis and xenobiotic metabolism (13, 33) can be highlighted as the two metabolic pathways altered by dietary soybean products. Downregulation of hepatic cytochrome P450s and other drug-metabolizing enzymes are linked to inflammation (58). Furthermore, CYP1A1, an enzyme in the phase I of the reactions which helps in the excretion of endogenous (*e.g.* steroids) and exogenous (*e.g.* drugs) substances (59), was lower in the colonic enterocytes of patients suffering from Crohn’s disease and ulcerative colitis (60). Our observation on Atlantic salmon also corroborates with this finding. The biosynthesis of steroids was enriched in the SO group, and the enrichment of two cytochrome P450-associated pathways could be indicating the need to alleviate the toxicity of steroids and soy saponin.

Hypotaurine is essential for the biosynthesis of the abundant free amino acid taurine, which has roles in innate immunity of mammals (61). High levels of this semi-essential amino acid reduce oxidative stress due to its antioxidant properties (62). In humans, taurine and *β*-alanine transport in the intestine takes place with the help of transporters like Na^+^- and Cl^−^-dependent TauT (SLC6A6) (56). Taurine is linked to growth and health of fish (63). A gene that is related to biosynthesis of taurine, *csad*, was upregulated in the distal intestine of fish from the SO group. It seems that *slc6a6*, *csad*, taurine, and hypotaurine metabolism pathways are associated with the inflammatory condition.

### Dietary Soy Components Affect Blood Lymphocyte Counts

The head kidney is the major hematopoietic organ in teleost fish, having roles similar to those of the mammalian bone marrow. Hematopoietic stem cells in the organ are capable of self-renewal and differentiating into cells of erythroid, myeloid, and lymphoid lineages (64). Injuries during inflammation or metabolic changes are known to influence the size and fate of hematopoietic stem cells (65). It has also been reported that systemic response during inflammation increased the number of granulocytes and monocytes in mice bone marrow (66). In the present study, we observed a significant increase in blood lymphocyte-like cell counts in fish fed the inflammation-causing soybean products compared to those of control. It is reported that in humans, the patrolling naive lymphocytes enter the gut and undergo activation and priming based on the condition in the intestine (67). The increase in blood lymphocyte counts of fish that developed inflammation is possibly indicative of the cell recruitment to quell the localised intestinal inflammation.

## Conclusions

The soybean–soy saponin combination induced DI inflammation in Atlantic salmon. The transcriptional changes associated with inflammation could be linked to disturbances in transport mechanisms as well as drug and taurine and hypotaurine metabolisms and steroid biosynthesis in the DI of the fish. The HK transcriptome was largely unaffected during intestinal injury. However, this needs to be verified as the increased lymphocyte numbers in the peripheral blood of the fish with intestinal inflammation may in fact be suggesting otherwise. Further investigations on the dysregulated intestinal barrier functions, reported here, will help to broaden our understanding of the intestinal inflammation in fish. The key genes involved in solute transport across the epithelial barrier such as *clcn1*, *clcn2*, *slc26a6*, *slc6a19* and immune genes such as *cath1*, *cath2*, *gal3*, *rbp2*, *mta* could serve as possible biomarkers of diet-associated intestinal inflammation and be used in further comparative studies on inflammation in mammalian models.

## Data Availability Statement

The datasets presented in this study can be found in online repositories. Additional data and figures are presented in **Supplementary Material**.

## Ethics Statement

The animal study was reviewed and approved by the Norwegian Animal Research Authority, FDU (Forsøksdyrutvalget ID-10050). We adhered to the rules and regulations of animal welfare and followed the standard biosecurity and safety procedures at the Research Station of Nord University, Bodø, Norway.

## Author Contributions

VK, VVT, JF, MS, and JD designed the research. JD and VVT were responsible for feed formulation and development. GV supervised the feeding experiments and gene expression study. YP and PS performed the RNA-Seq studies and analyses of data. YP performed the flow cytometry studies. DD performed the histology study. VK wrote the first version of the manuscript, along with YP and PS. All authors contributed to the article and approved the submitted version.

## Funding

This study was undertaken as part of a collaborative project “Molecular studies on the intestine of Atlantic salmon” between Nord University and DSM Nutritional Products, Switzerland, funded by the latter.

## Conflict of Interest

JD is employed by the company SPAROS Lda. Olhão, Portugal. VVT was employed by the company DSM Nutritional Products, Global Innovations, Kaiseraugst, Switzerland.

The remaining authors declare that the research was conducted in the absence of any commercial or financial relationships that could be construed as a potential conflict of interest.

The authors declare that this study received funding from DSM Nutritional Products. The funder had the following involvement in the study: research design and feed formulation.

## Supplementary Material

Supplementary Figure 1 Photomicrographs of the distal intestine of Atlantic salmon from CO and SO groups. **(A)** Inflammatory symptoms are not visible in the SO group on day 4 after the start of the feeding trial. **(B)** Saponin-induced inflammatory characteristics become visible at day 36 after the start of the feeding. Control group—CO, and soy-derivatives fed group—SO. Scale bar: 100 µm.

Supplementary Figure 2 Overview of the bioinformatics workflow adopted in this study. RNA-Seq data were first quality filtered, after which the reads were aligned to salmon genome. Next, the read counts were extracted, and then upregulated/downregulated genes were identified for the downstream analysis. KEGG pathway and GO term enrichment analysis were then performed employing the differentially expressed genes.

Supplementary Figure 3 Dispersion estimates and minus over average expression plots of the data from the distal intestine. **(A)** Plots showing the shrinkage of the gene-wise dispersions—the maximum-likelihood estimates obtained from the gene data (black) are shrunk towards the fitted red curve to get the final values (blue) of dispersion. **(B)** Shrinkage of the logarithmic fold-change—the differentially expressed genes with adjusted p < 0.05 are shown as red dots.

Supplementary Figure 4 Dispersion estimates and minus over average expression plots based on the data from the head kidney. **(A)** Plots showing the shrinkage of the gene-wise dispersions—the maximum-likelihood estimates obtained from the gene data (black) are shrunk towards the fitted red curve to get the final values (blue) of dispersion. **(B)** Shrinkage of the logarithmic fold-change—the differentially expressed genes with adjusted p < 0.05 are shown as red dots.

Supplementary Figure 5 Heatmap showing the 53 upregulated and 38 downregulated genes in the distal intestine of Atlantic salmon. Normalized gene count was input into the pheatmap function, and the values were centered and scaled in the row direction. The column names at the bottom of the heatmap indicate the control (prefix CO) and the soybean-derivative (saponin group with prefix SO) fed groups.

Supplementary Figure 6 Relative mRNA levels of the selected 15 genes in the distal intestine of Atlantic salmon from the CO and SO groups at day 36. The gene expression of control (CO) and soybean fed groups (SO) (n = 6) is shown. Asterisks indicate **p < 0.01 and ***p < 0.001.

Supplementary Figure 7 Correlation between the relative mRNA levels and read counts of the selected differentially expressed genes. The bar on the right side indicates the correlation coefficient; darker shades of red and blue indicate negative and positive correlations, respectively. X indicates non-significant differences based on Spearman correlation test. If the correlation coefficient is + or −0.75, 3/4^th^ of the circles will be shaded.

Supplementary Table 1 Details of ingredients in the experimental feeds.

Supplementary Table 2 Details of raw reads, cleaned reads and mapped reads from different samples.

Supplementary Table 3 Details of differentially upregulated genes in the SO group compared to the CO group.

Supplementary Table 4 Details of differentially downregulated genes in the SO group compared to the CO group.

Supplementary Table 5 Details of differentially regulated genes in the SO group compared to the CO group.
