# Supplementary material for: Intestinal Transcriptome Analysis Reveals Soy Derivative-Linked Changes in Atlantic Salmon
Source: Front Immunol. 2020 Dec 11;11:596514. doi: 10.3389/fimmu.2020.596514 (PMC7759687; doi:10.3389/fimmu.2020.596514)

Supplementary Figure 1

A

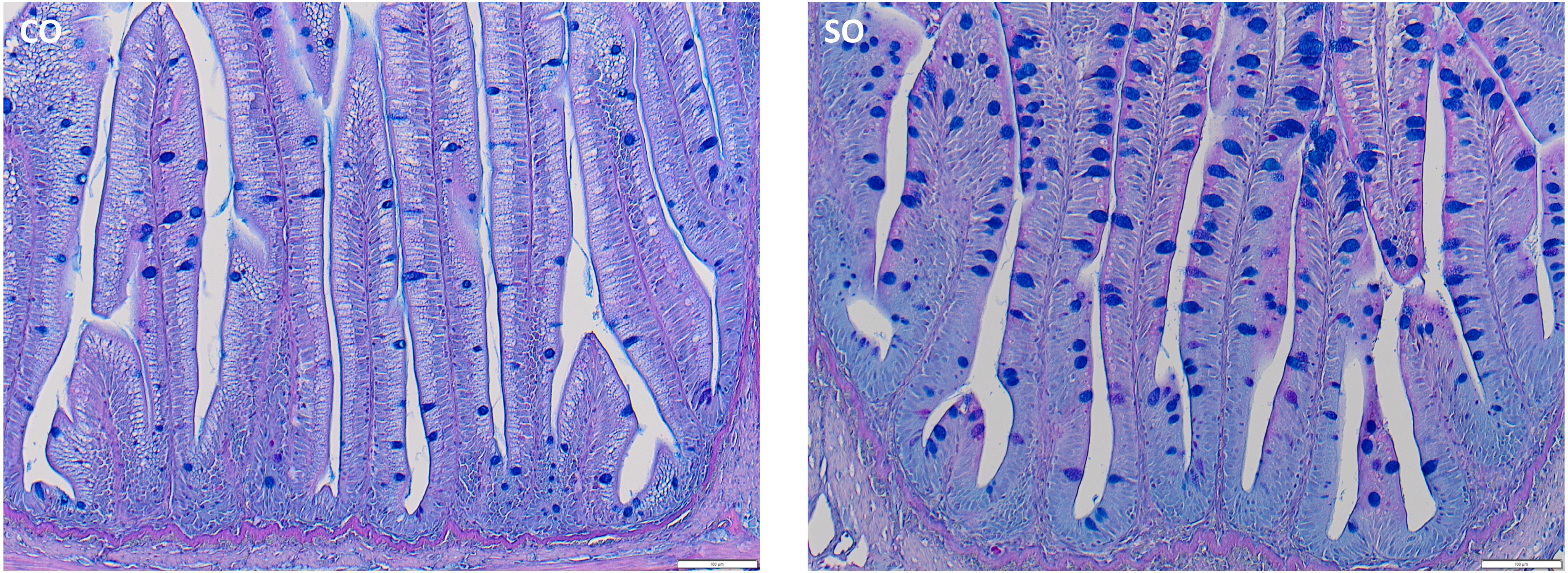

Supplementary Figure 1

B

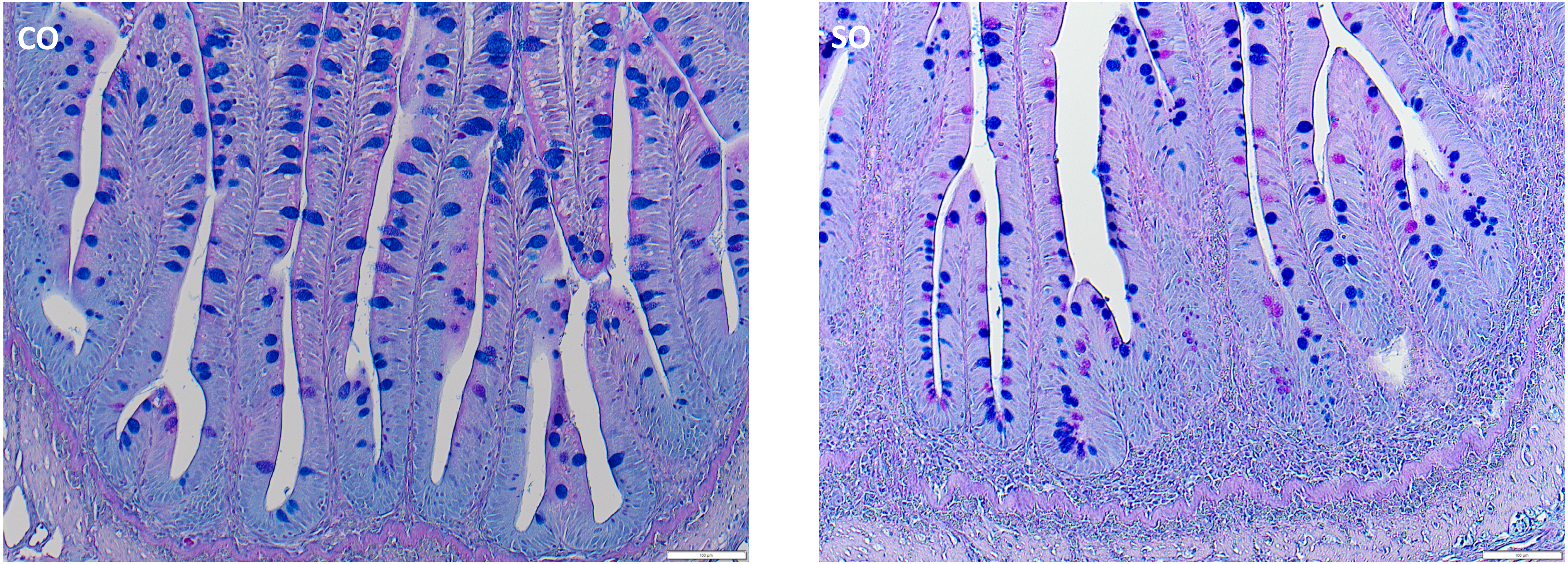

Supplementary Figure 2

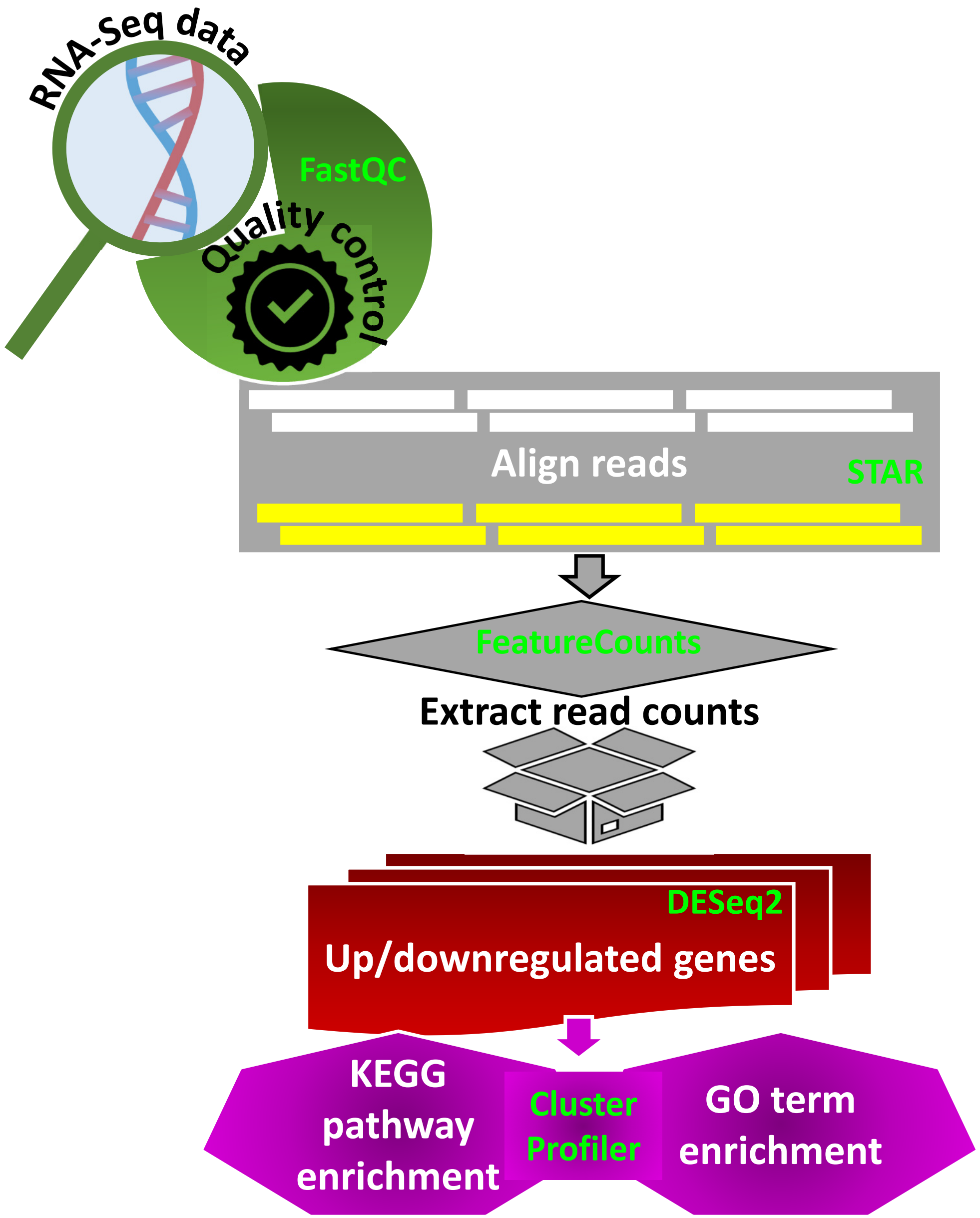

Supplementary Figure 3

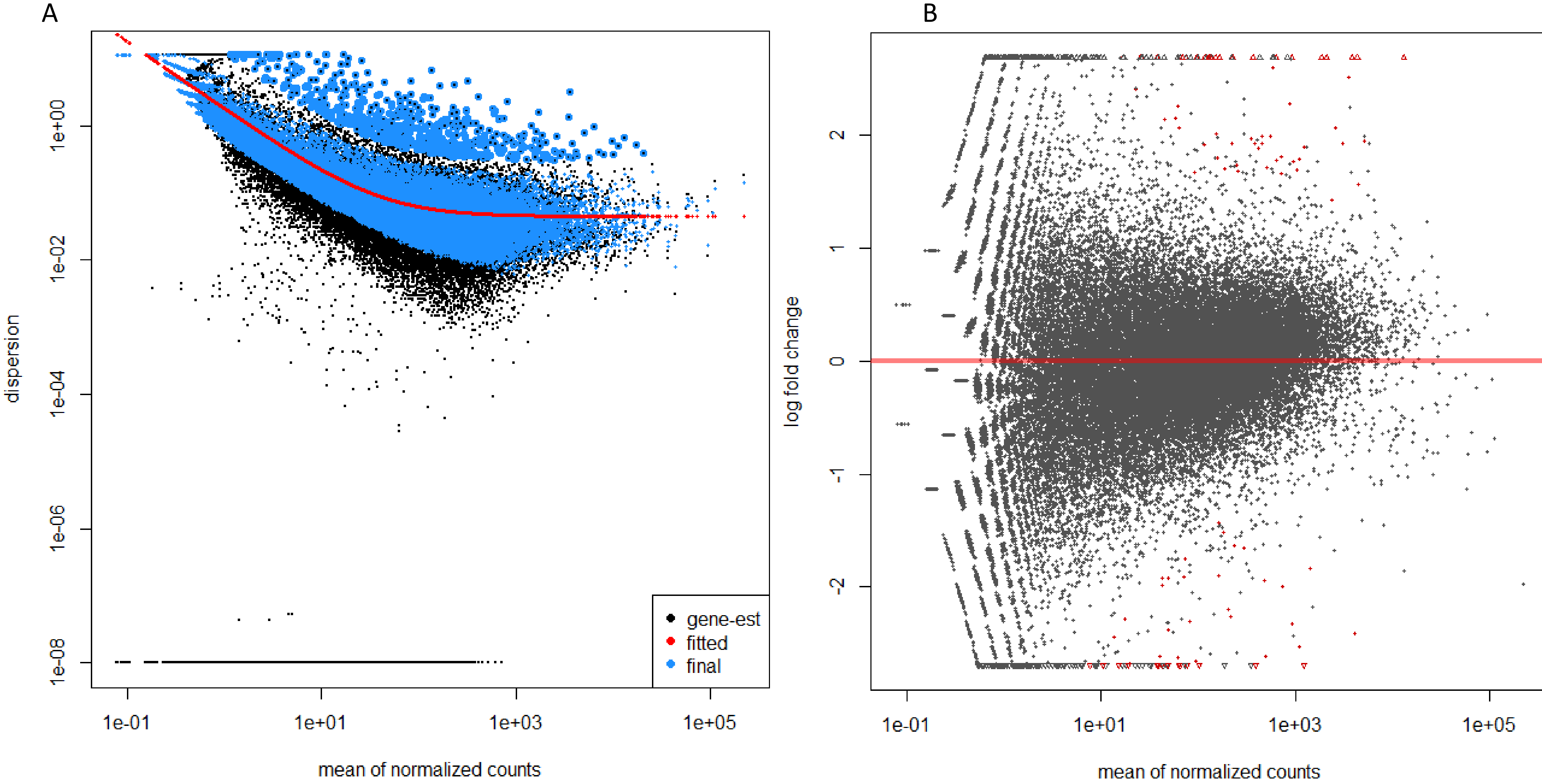

Supplementary Figure 4

A

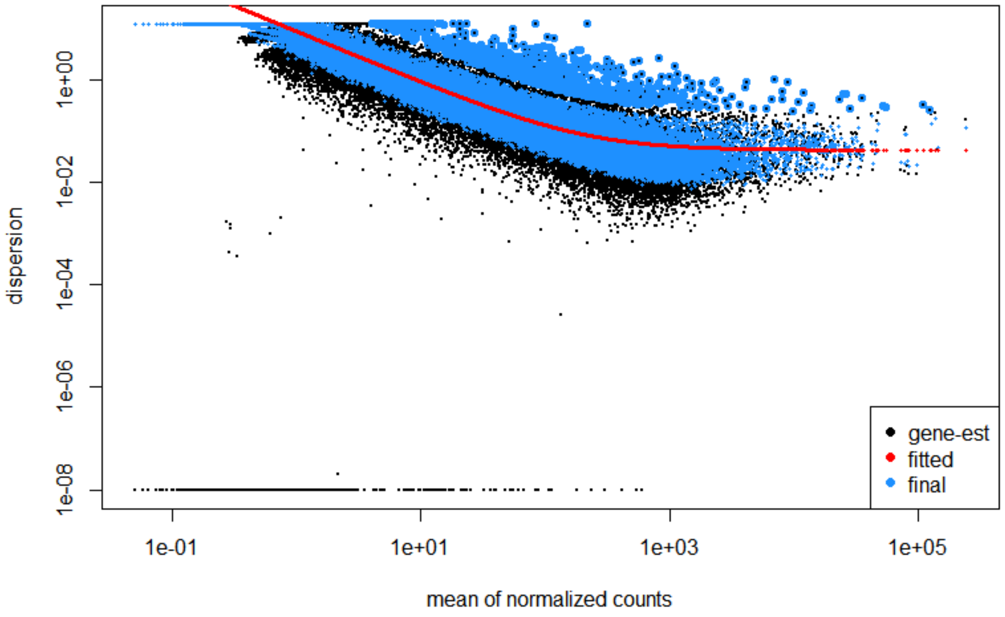

B

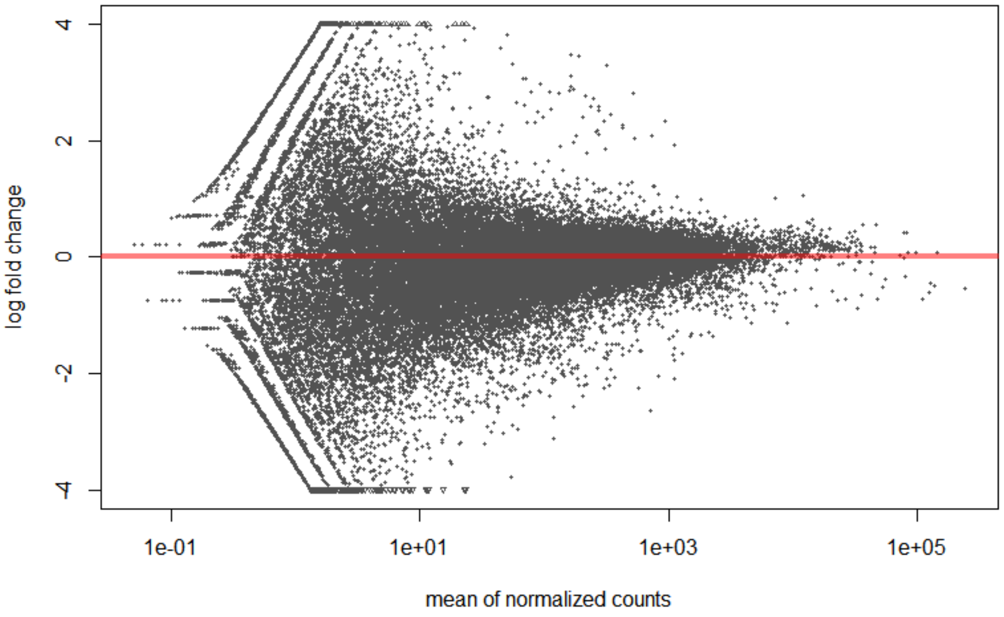

Supplementary Figure 5

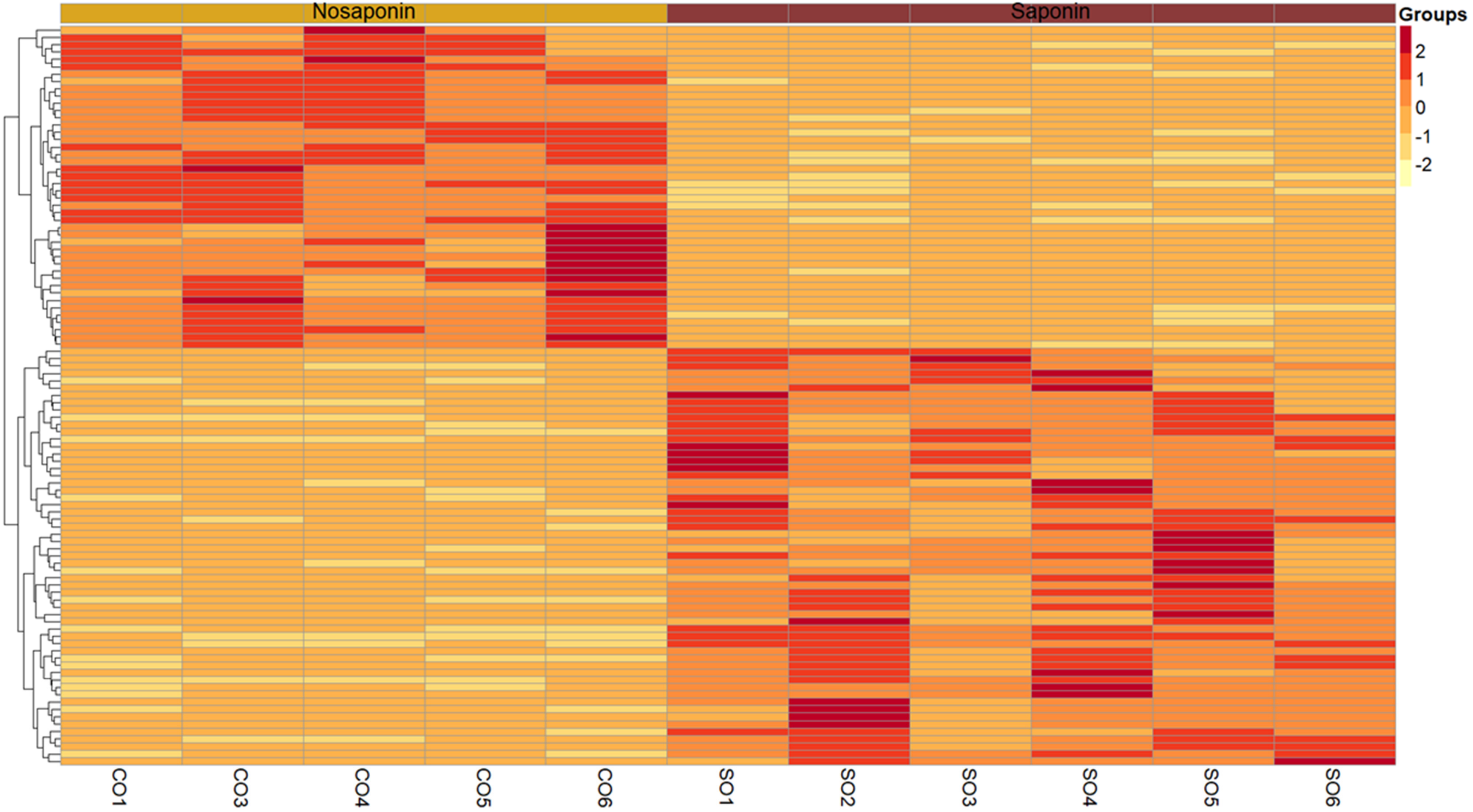

Supplementary Figure 6

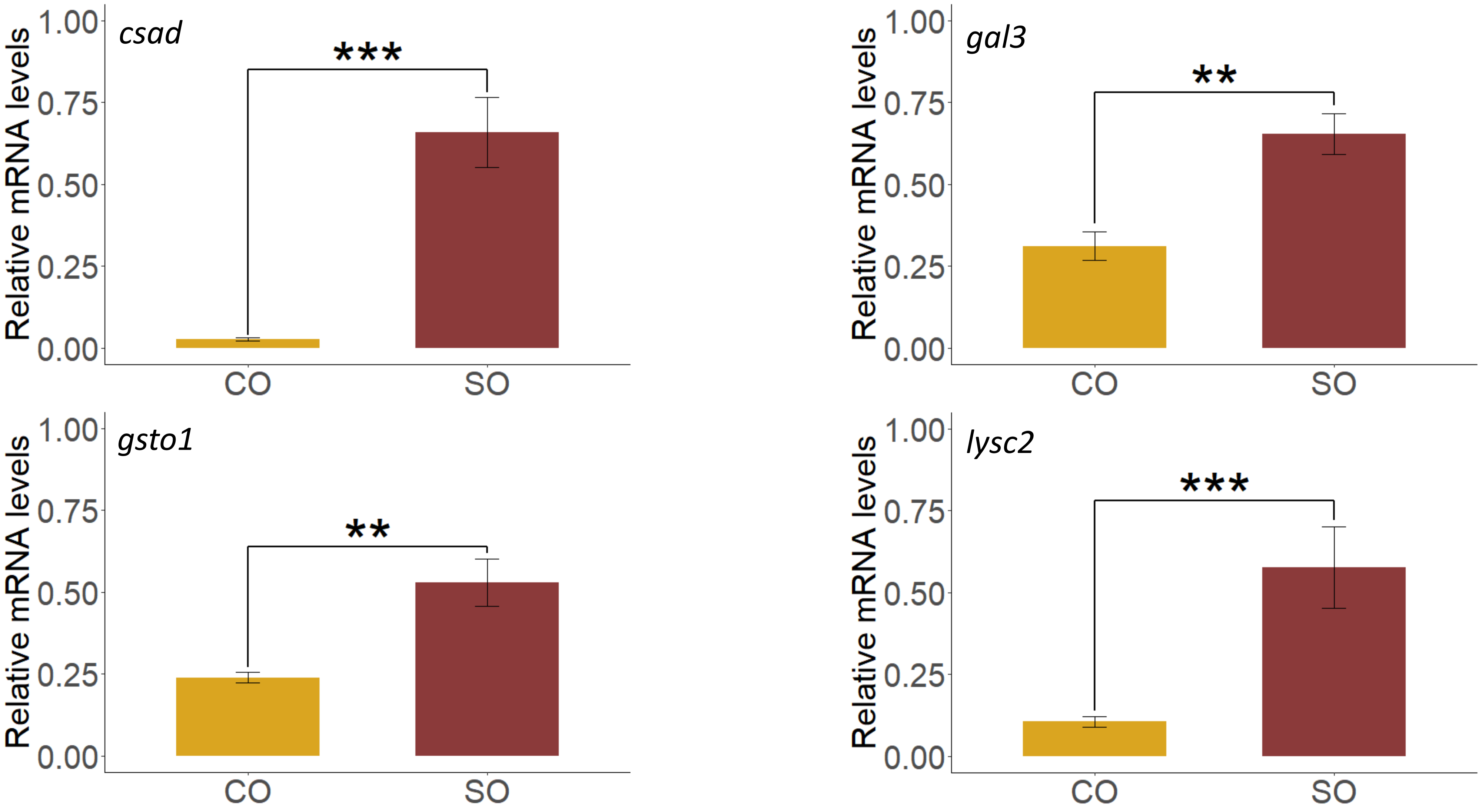

Supplementary Figure 6

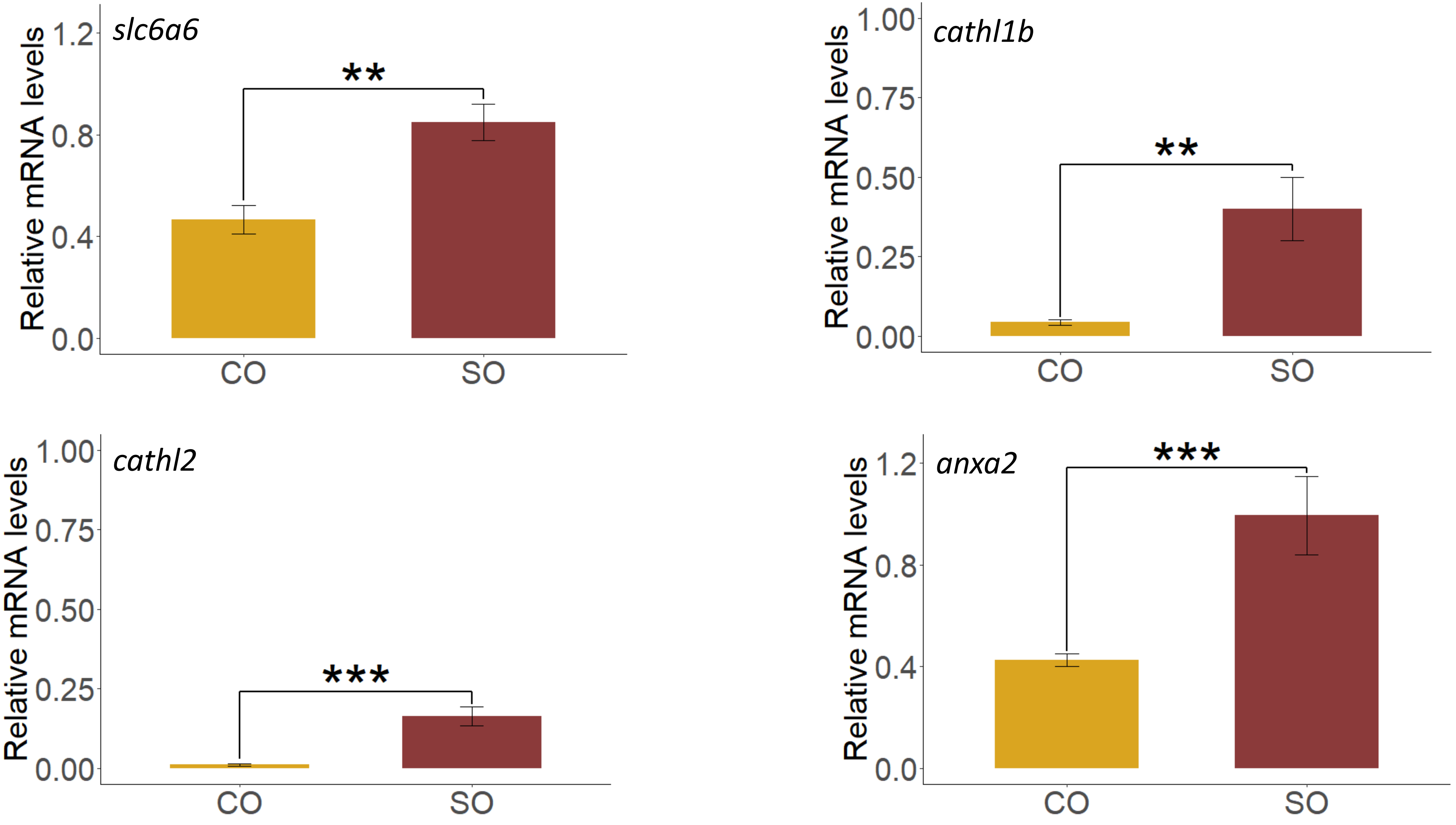

Supplementary Figure 6

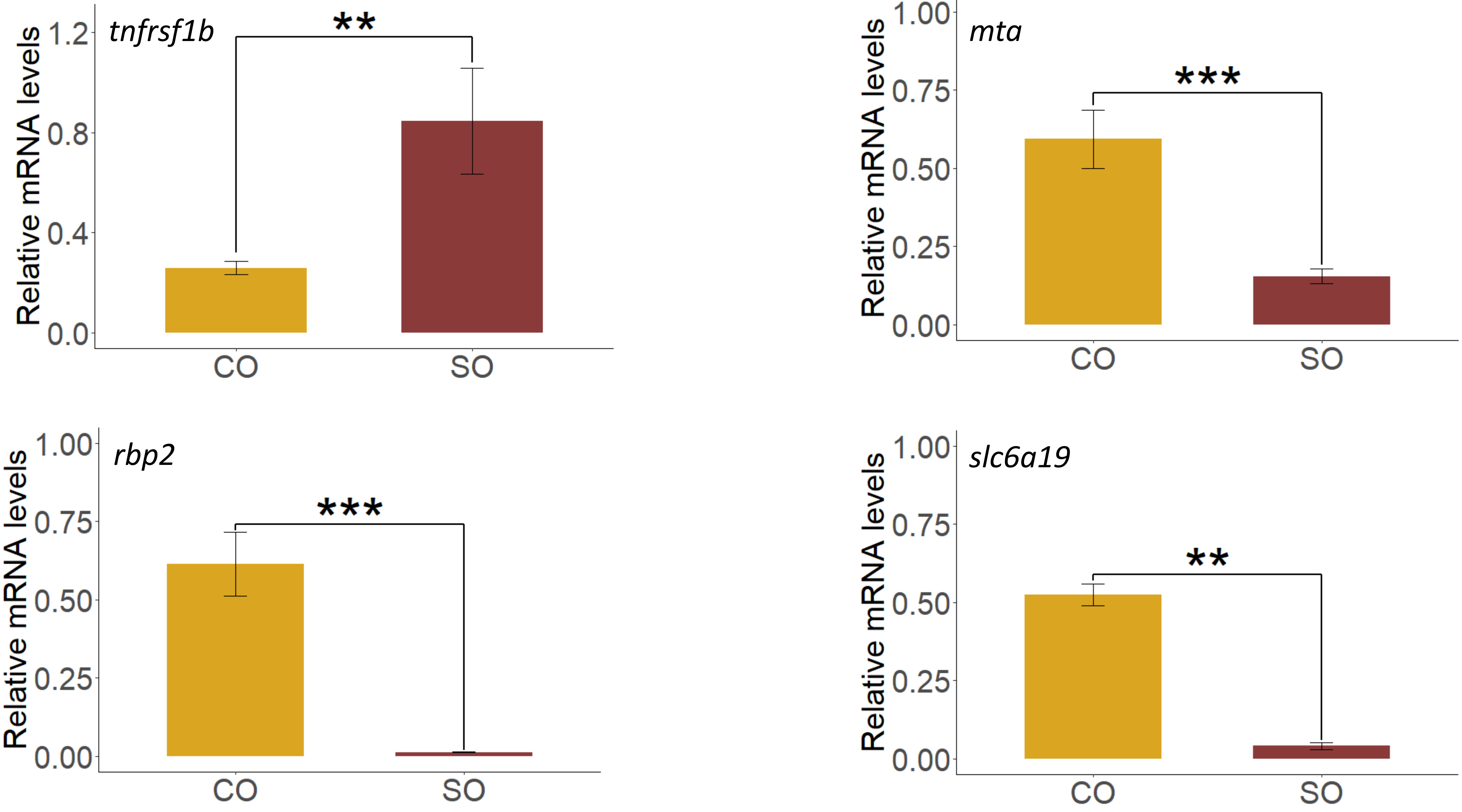

Supplementary Figure 6

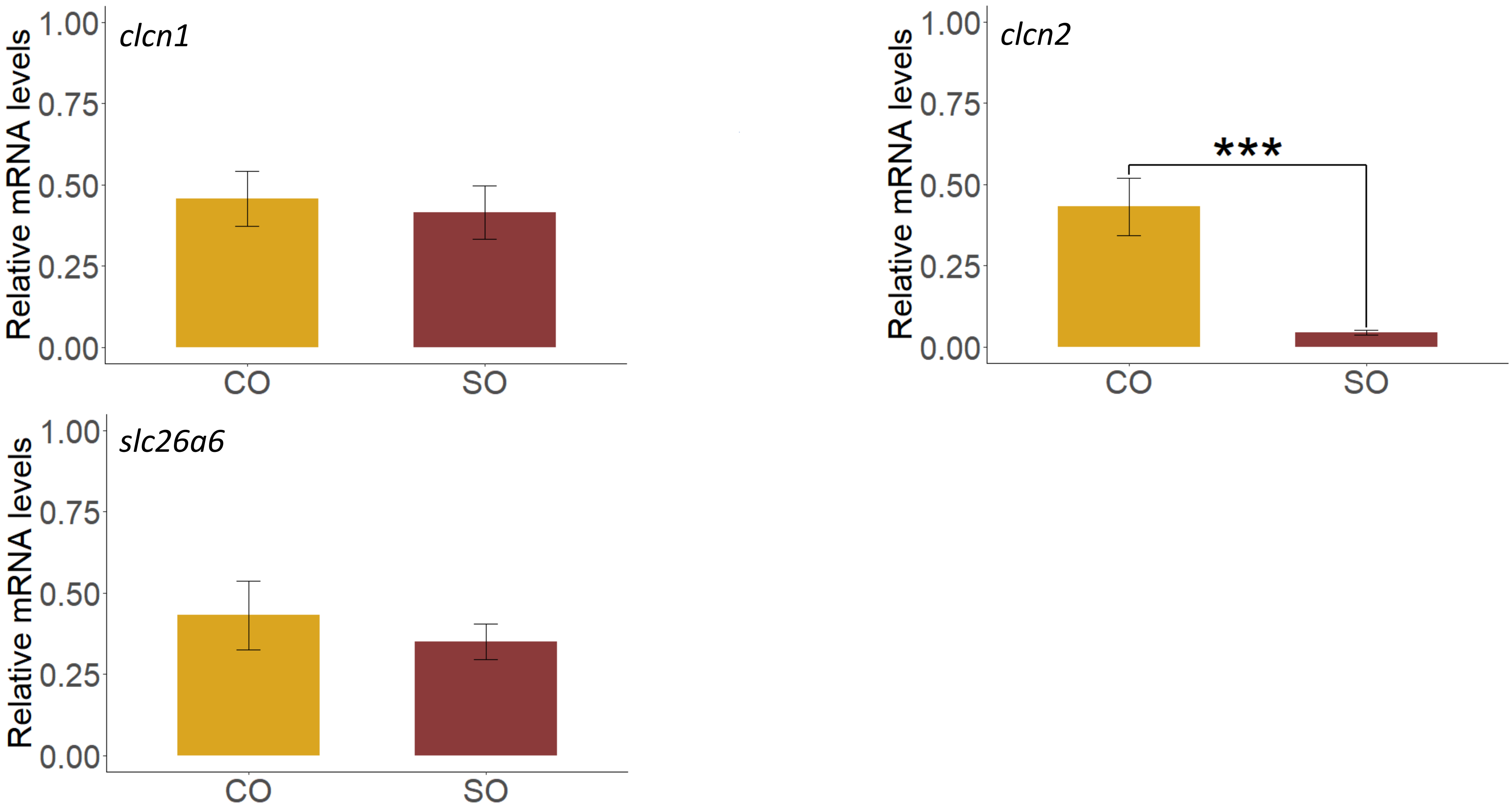

Supplementary Figure 7

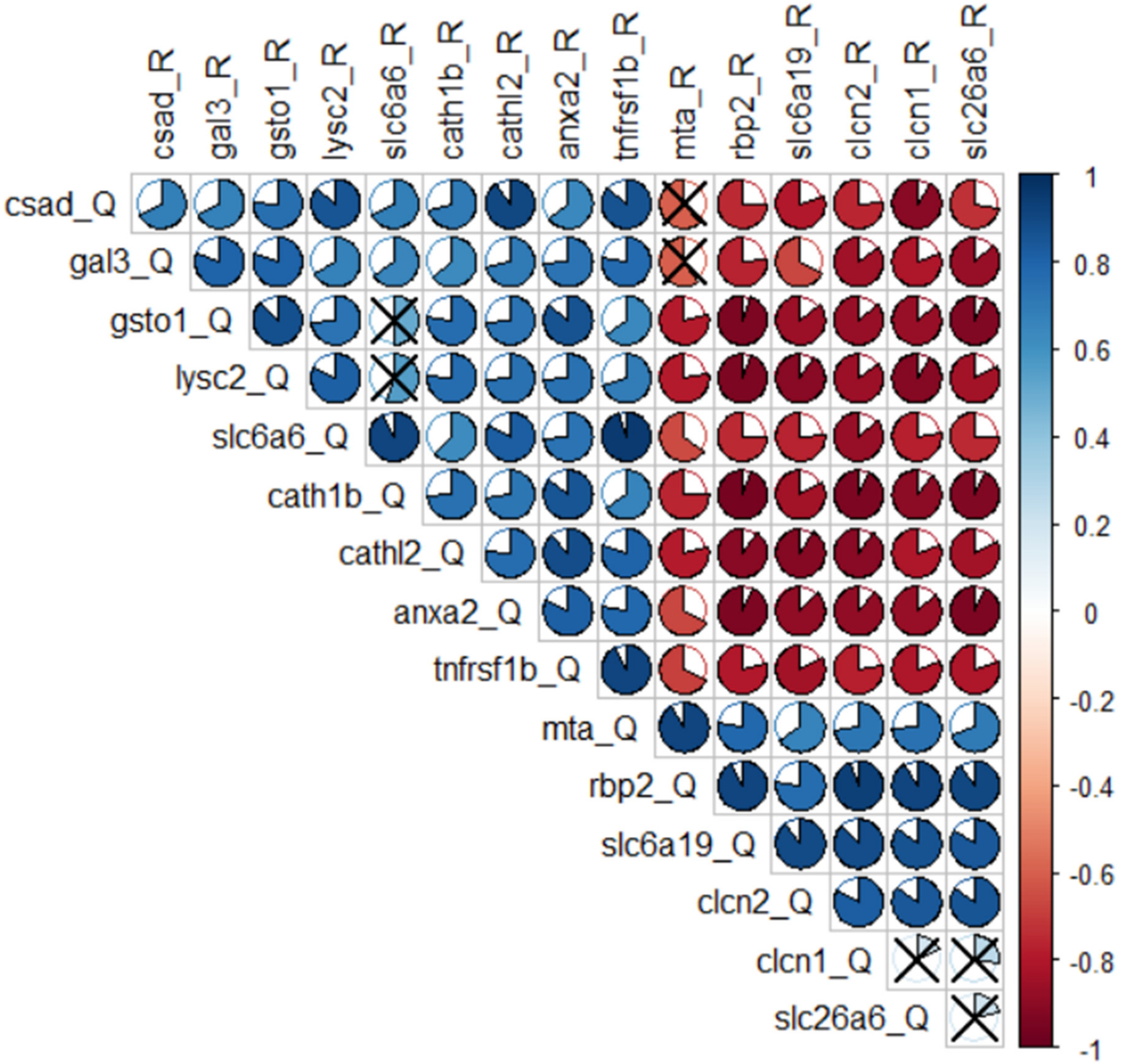

Supplement: Supplementary Figure 1 — Photomicrographs of the distal intestine of Atlantic salmon from CO and SO groups. (A) Inflammatory symptoms are not visible in the SO group on day 4 after the start of the feeding trial. (B) Saponin-induced inflammatory characteristics become visible at day 36 after the start of the feeding. Control group—CO, and soy-derivatives fed group—SO. Scale bar: 100 µm. [file DataSheet_1.pdf]
